# The influence of social support on active aging in chronic disease patients among older adults: mediating role of coping strategies

**DOI:** 10.3389/fpubh.2026.1747285

**Published:** 2026-01-27

**Authors:** Chengping Jian, Xiangdong Peng, Haiyan Tian, Qian Ding

**Affiliations:** 1 Department of Ophthalmology, Mianyang Central Hospital, Mianyang, China; 2 Department of Cardiothoracic Surgery, Mianyang Central Hospital, Mianyang, China; 3 Department of Nursing, Yuxi People's Hospital, Yuxi, China

**Keywords:** active aging, chronic disease patients, older adults, mediating role, social support

## Abstract

**Background:**

Active aging is pivotal for addressing population aging. Older adults chronic disease patients face greater active aging challenges, and while social support and coping strategies correlate with it, their mediating mechanism in this population remains underverified. This study investigated the effect of social support on the level of active aging among older adults with chronic diseases and further examined whether coping styles play a mediating role in this relationship.

**Methods:**

Between August 2024 and May 2025, patients were surveyed using the Chinese versions of the Active Aging Scale, the Medical Coping Modes Questionnaire, and the Social Support Questionnaire. In addition, demographic information was collected, and statistical analyses were conducted to evaluate the data. Correlation analyses and mediation effect tests were performed using SPSS 25.0 and Amos 24.0, respectively.

**Results:**

The results showed that social support was significantly and positively associated with both active aging (*r* = 0.422, *p* < 0.01) and medical coping styles (*r* = 0.408, *p* < 0.01). In addition, medical coping styles were strongly and positively correlated with active aging (*r* = 0.485, *p* < 0.01). Mediation analysis further demonstrated that medical coping styles partially mediated the relationship between social support and active aging. Specifically, the direct effect of social support accounted for 71.72% of the total effect, whereas the indirect effect transmitted through coping styles explained the remaining 28.28%. Among the coping dimensions, the confrontation coping style exerted a significant positive influence on active aging (*β* = 0.474, *p* < 0.001).

**Conclusion:**

Social support contributes to higher levels of active aging among older adults with chronic diseases through both direct and indirect pathways. In addition to its direct influence, social support indirectly promotes active aging by encouraging patients to adopt a confrontation-oriented coping style. These findings indicate that coping style functions as a key mediating mechanism linking social support to active aging in older adults individuals with chronic conditions.

## 1 Introduction

Population aging has become a significant global challenge in social development. Statistics indicate that from 2000 to 2050, the proportion of the global population aged 60 years and older will rise from 10 to 21%. In China, this proportion is expected to increase from 10 to 33.3%, with individuals aged 65 years and older accounting for 26.9% of the total Chinese population by 2050 (1). As one of the countries with the highest number of older adults population, China’s population aging is marked by rapid progression, a large affected population, and increasing depth, placing substantial pressure on the national healthcare system (2). As population aging accelerates and multimorbidity becomes more prevalent among older adults, the health status, overall well-being, and quality of life of older adults individuals with chronic diseases have emerged as central issues of societal concern (3). According to the World Health Organization(WHO) (4), active aging is defined as a process in which older adults maximize opportunities for health, participation, and security to enhance their quality of life. It also refers to older adults maintaining good physical, social, and psychological states throughout the life course and actively facing later life. Active aging serves as a comprehensive framework for evaluating quality of life in older adults, encompassing not only the maintenance of physical health but also broader dimensions such as social engagement and self-realization. For older individuals living with chronic conditions, fostering active aging involves maintaining autonomy in daily activities, sustaining meaningful social relationships, and effectively managing long-term illnesses. The realization of these goals is closely associated with the sustainable development of societies undergoing demographic aging.

Therefore, identifying the determinants and underlying mechanisms of active aging has significant practical implications for the construction of age-friendly social support systems and for improving the overall well-being of older adults. Social support is a crucial external factor for older adults patients with chronic diseases to alleviate disease-related stress and sustain daily living capacities. It positively assists patients in adapting to their illness and maintaining self-care capabilities (5). Emotional support derived from family caregiving, service-related assistance provided by community-based healthcare systems, and resource support accessed through broader social networks jointly promote active aging by reducing psychological distress, improving adherence to treatment regimens, and alleviating disease-related social isolation (6, 7). Social support improves health literacy and self-efficacy among older adults patients, thereby forming a foundation for selecting and implementing coping strategies. Magi et al. (8), in their systematic review on health literacy and self-care in chronic disease patients, proposed that coping decisions by older adults chronic patients are essentially self-regulated behaviors driven by self-efficacy. Self-efficacy, defined as the individual’s confidence in managing disease-related tasks, directly influences the choice and effectiveness of coping strategies. Patients with high self-efficacy are more likely to adopt proactive coping methods, a process that requires sufficient health literacy. Additionally, social resource support provides external collaboration conditions for executing coping strategies, such as participation in mutual support groups (9). Effective implementation of coping strategies further promotes core behaviors of patient engagement in chronic disease self-management, including dietary adjustments and lifestyle changes to manage their conditions (10). Social coping strategies, such as active communication and mutual participation, help maintain social connections. Ultimately, these behaviors facilitate active aging by maintaining physical health and expanding social participation. This perspective is consistent with the findings of Li et al. (11), who demonstrated that coping strategies mediated the relationship between social support and depression (a core indicator of passive aging) among older adults patients with hypertension. Active coping strategies strengthened the positive effect of social support on self-management behaviors, thereby reducing the risk of depression.

Coping styles, defined as the cognitive and behavioral strategies individuals employ to manage stressful situations, are critical determinants of disease adaptation and quality of life among older adults with chronic conditions. Positive coping strategies, such as actively seeking medical information, adjusting one’s mindset to cooperate with treatment, and participating in peer support groups, can strengthen the beneficial effects of social support (12). In contrast, maladaptive coping behaviors, including avoidance of illness-related concerns, social withdrawal, and resistance to intervention, may weaken the effectiveness of available support resources and thus act as barriers to active aging (13). Existing research has largely concentrated on the direct relationship between social support and active aging (9) or has examined the influence of coping styles on adaptation outcomes in patients with chronic diseases in isolation (14). However, the mechanisms linking these three constructs remain insufficiently explored. In particular, it is still unclear whether social support indirectly affects active aging among older adults with chronic diseases by shaping their coping strategies, and the magnitude and structural pathway of this potential mediating effect have yet to be fully elucidated.

This study targets older adults with chronic diseases, investigating the relationship chain of “social support → coping strategies → active aging.” It aims to clarify the mediating role of coping strategies in the link between social support and active aging. By doing so, the research seeks to promote higher levels of active aging among older adults individuals with chronic conditions and to provide empirical evidence for the development of precision-oriented management and support systems tailored to this population.

## 2 Method

### 2.1 Study design and sample

Data were collected from older adults with chronic diseases who attended Yuxi People’s Hospital between August 2024 and May 2025. Patients meeting the inclusion criteria were invited to complete a structured questionnaire, including the following measures: (a) aged 60 years or older; (b) diagnosed with at least one chronic disease by a medical institution; and (c) no cognitive impairment, clear consciousness, and able to complete the questionnaire independently or with assistance. Patients experiencing acute exacerbation of diseases requiring immediate emergency care were excluded.

### 2.2 Measures

#### 2.2.1 Demographic characteristics

The study collected demographic and lifestyle information, including age, gender, marital status, educational attainment, sources of financial support, family circumstances, and personal lifestyle habits. Age was categorized into three groups: 60–69, 70–79, and 80 years or older. Marital status was classified as unmarried, married, or widowed. Educational attainment was divided into five levels: below junior high school, high school or vocational school, college diploma, bachelor’s degree, and graduate degree or higher. Primary sources of financial support included assistance from children, retirement pensions, or personal income from employment.

#### 2.2.2 Chinese version of the active aging scale, AAS

The study employed the Chinese version of the Active Aging Scale, translated by Zhang et al. (15). This 36-item instrument assesses seven dimensions of active aging: self-care, active learning and social integration, economic stability, spiritual development, healthy living, societal contribution, and filial piety. Responses are recorded on a 4-point Likert scale, with higher scores indicating greater levels of active aging. The Cronbach’s *α* coefficients for the seven dimensions were 0.905, 0.871, 0.721, 0.784, 0.788, 0.806, and 0.749, respectively.

#### 2.2.3 Medical coping modes questionnaire, MCMQ

The Chinese version of the Medical Coping Style Questionnaire, revised by Shen et al. (16), was used to assess patients’ coping strategies. This 20-item instrument includes three dimensions: confrontation, acquiescence, and avoidance, and responses are recorded on a 4-point Likert scale. Eight items are reverse-scored, and higher scores within each dimension indicate a stronger tendency toward that coping style. The Cronbach’s *α* coefficients for the three dimensions were 0.843, 0.824, and 0.816, respectively.

#### 2.2.4 Social support rating scale, SSRS

The Social Support Questionnaire, developed in 1994 by Professor Xiao Shuiyuan and colleagues in China (17), consists of 10 items across three dimensions: support utilization, subjective support, and objective support. Items 1–4 and 8–10 are rated on a 4-point Likert scale (1–4), while Item 5 uses a 5-point Likert scale (0–4), with “None” scored as 0 and “Strongly Agree” scored as 4. Items 6 and 7 are scored according to the number of sources selected, with “No sources” scored as 0 and “The following sources” scored based on the total sources indicated. Total scores range from 12 to 66, with higher scores reflecting greater levels of social support: 12–21 indicates low support, 22–44 moderate support, and 45–66 high support. The Cronbach’s *α* coefficients for the three dimensions were 0.703, 0.792, and 0.727, respectively.

### 2.3 Data collection

Data were collected using a combination of online and offline methods. All surveys were conducted anonymously, and participants were assured that their personal information would remain strictly confidential. For online data collection, patients who could access the questionnaire via QR codes completed the survey on “WenJuanXing,” a Chinese online survey platform. QR codes were distributed through WeChat, and system settings restricted each participant to a single submission to prevent duplicate responses. After data collection, researchers exported the responses from the platform into Excel. Invalid responses, defined as surveys completed in under five minutes or showing inconsistent or non-random answering patterns, were excluded from analysis.

For patients unable to access the online questionnaire, offline surveys were administered. After obtaining informed consent, researchers distributed printed questionnaires and provided standardized instructions regarding completion procedures and key considerations. Participants were encouraged to complete the questionnaires independently; however, for those with physical or cognitive limitations, verbal assistance was provided while ensuring that responses accurately reflected the participants’ own views. To maintain data integrity, each questionnaire was checked for missing items immediately upon submission. A total of 355 questionnaires were distributed.

### 2.4 Statistical analysis

Data were analyzed using SPSS 25.0 and AMOS 24.0. The analytical process began with tests for common method bias and assessment of normality using the Shapiro–Wilk test. Categorical variables are presented as n (%), while continuous variables with non-normal distributions are described using medians and interquartile ranges. Group comparisons were performed using the Mann–Whitney U test or the Kruskal-Wallis H test, as appropriate. Pearson correlation analysis was employed to examine relationships between variables. Structural equation modeling was conducted in AMOS 24.0, with model validation performed using the Bootstrap method. The criteria for evaluating model fit were defined as follows: Chi-square to degrees of freedom ratio (χ^2^/df) < 3, Root Mean Square Error of Approximation (RMSEA) < 0.08, Goodness-of-Fit Index (GFI) > 0.9, Normed Fit Index (NFI) > 0.9, Tucker-Lewis Index (TLI) > 0.9, Incremental Fit Index (IFI) > 0.9, and Comparative Fit Index (CFI) > 0.9. A two-sided significance level of *α* = 0.05 was applied for all statistical tests.

### 2.5 Ethical considerations

This study was approved by the Medical Ethics Committee of Yuxi People’s Hospital (ID: 2024kmykdx6f027). Electronic informed consent was obtained at the start of the online questionnaire, requiring participants to actively opt in; the survey automatically terminated if consent was not provided and proceeded only upon agreement. For offline participants, written informed consent was obtained prior to participation. Enrollment in the study commenced only after consent was given, and all participants were informed of their right to withdraw from the study at any time without penalty.

## 3 Results

### 3.1 Characteristics of participants

A total of 355 questionnaires were distributed, comprising 50 paper-based and 305 electronic surveys. Following data screening, 54 invalid questionnaires were excluded, yielding 301 valid responses for analysis. Among the 301 participating chronic disease patients, the highest proportions were hospitalized for circulatory system diseases (50.50%) and respiratory system diseases (40.53%). Additionally, 178 patients (59.1%) had two or more chronic diseases. Analysis revealed significant differences (*p* < 0.05) in active aging scores among chronic disease patients across various demographics: age, healthcare access methods, marital status, monthly personal income, economic sources, number of children, housing type, employment status, and sleep patterns. Significant differences in medical coping scores were also observed across patients of different ages, marital statuses, and employment statuses (*p* < 0.05). As shown in Table 1, patients’ social support scores exhibited significant variations (*p* < 0.05) based on age, religious affiliation, marital status, monthly personal income, number of children, living arrangements, exercise habits, and sleep patterns.

**Table 1:** Sample characteristics (*n* = 301).

Item	Number (%)	Active aging score	Z/H	*p* value	Score of coping style	Z/H	*P* value	Social support score	Z/H	*P* value
Gender										
Male	139 (46.2)	100 (77, 116)	−0.401	0.688	56 (47, 67)	−0.649	0.516	34 (31, 38)	−0.552	0.581
Female	162 (53.8)	98 (74, 116)			55.5 (43, 65)			35 (29.75, 40)		
Age										
60–69	142 (47.2)	101 (77, 118)	17.715	0.001	55 (42, 63.25)	11.820	0.003	35 (31, 41)	6.586	0.037
70–79	95 (31.6)	104 (81, 116)			60 (49, 69)			35 (31, 38)		
≥80	64 (21.3)	81 (70, 99.75)			52.5 (44, 61.5)			33 (29, 36.75)		
Education background										
Junior high school (or technical school) and below	215 (71.4)	99 (77, 116)	4.777	0.311	57 (44, 67)	3.641	0.457	34 (30, 38)	7.651	0.105
High school/technical secondary school	49 (16.3)	97 (72, 110.5)			51 (43, 62)			34 (30, 37.5)		
Specialized subject in college	18 (6)	103 (77.5, 123.25)			54.5 (47, 62.25)			35 (28, 41)		
Bachelor degree	18 (6)	106.5 (86.75, 119.25)			56 (46.75, 63.25)			41 (34, 43.5)		
Postgraduate degree or above	1 (0.3)	74 (74, 74)			42 (42, 42)			33 (33, 33)		
Religious belief										
Yes	43 (14.3)	92 (70, 110)	−1.884	0.060	50 (41, 61)	−1.833	0.067	32 (28, 37)	−2.660	0.008
No	258 (85.7)	100 (77, 116)			57 (44.75, 66)			35 (31, 39)		
Medical consultation methods										
Resident medical insurance	212 (70.4)	97 (74.25, 111)	11.867	0.003	56 (44, 65)	3.524	0.172	34 (30, 38)	2.437	0.296
Employee medical insurance	69 (22.9)	109 (86, 119.5)			59 (46.5, 68)			36 (31, 40.5)		
Commercial insurance	20 (6.6)	78 (69, 110.75)			48 (41, 61.5)			33.5 (29.25, 37.75)		
Marital status										
Remarried/Divorced/Separated	244 (81.1)	102 (80, 116)	29.330	0.001	58 (47, 67)	20.532	0.001	35 (31, 39)	19.946	0.001
Unmarried	10 (3.3)	102.5 (87.5, 140)			50 (40.5, 61.25)			39.5 (26.25, 47.5)		
Widowed	47 (15.6)	72 (66, 98)			45 (38, 57)			31 (27, 35)		
Individual monthly income										
<1000	139 (46.2)	95 (72, 111)	10.249	0.006	54 (41, 64)	5.475	0.065	34 (30, 38)	6.965	0.031
1000–2000	88 (29.2)	100 (78.25, 115.75)			59 (47, 68)			34 (29, 37)		
≥2000	74 (24.6)	108 (81, 119)			55.5 (46.75, 66)			36 (31.75, 41)		
Source of income										
Child support	119 (39.5)	95 (72, 109)	8.135	0.017	57 (42, 65)	1.520	0.468	33 (30, 38)	2.902	0.234
Pension/Retirement insurance	98 (32.6)	103.5 (79.5, 117.25)			55.5 (47, 66.25)			35 (30, 39)		
Individual labor income	84 (27.9)	100 (85, 116)			54.5 (44, 64)			35 (30.25, 40)		
Number of children										
1–2	143 (47.5)	103 (80, 116)	9.136	0.028	56 (47, 64)	7.672	0.053	35 (31, 41)	9.812	0.020
3–5	109 (36.2)	96 (70.5, 111)			57 (44, 68)			34 (30, 38)		
More than 6	29 (9.6)	99 (81, 113)			54 (41.5, 69)			34 (33, 36)		
None	20 (6.6)	82 (67.5, 111)			46 (32, 59.5)			30 (28, 33.5)		
Form of residence										
Living with children and spouse	149 (49.5)	97 (71.5, 111)	10.885	0.020	55 (44, 65)	2.686	0.261	34 (29.5, 38.5)	31.582	0.001
Living with spouse	108 (35.9)	104.5 (85, 118.75)			57.5 (47.25, 66.5)			36 (32.25, 41)		
Living alone	44 (14.6)	90.5 (74, 109.25)			48.5 (40, 68)			31 (27.25, 33.75)		
Exercise habit										
None	80 (26.6)	92.5 (70.25, 114)	1.985	0.576	54 (41, 66.5)	2.336	0.506	32.5 (29, 36)	16.035	0.001
1–2 times per week	88 (29.2)	100 (79.25, 114.75)			57 (47, 66.75)			34 (31, 38)		
3–5 times per week	56 (18.6)	99.5 (77.25, 116)			55.5 (44, 66)			36 (32, 40.75)		
Exercise every day	77 (25.6)	100 (74.5, 116)			56 (43.5, 64)			37 (30, 41)		
Employment situation										
Retirement	172 (57.1)	103 (81, 118)	22.644	0.001	59 (47.25, 68.75)	20.802	0.001	34 (31, 38)	0.047	0.977
Currently employed	34 (11.3)	103.5 (73.75, 119.5)			48.5 (35.75, 61.25)			34.5 (29, 38.75)		
Sole proprietorship	95 (31.6)	86 (70, 103)			51 (41, 60)			35 (30, 40)		
Sleep status										
Extremely terrible	37 (12.3)	99 (75.5, 116.5)	16.364	0.001	57 (44, 68)	2.546	0.467	30 (28, 35)	22.485	0.001
Terrible	93 (30.9)	85 (69, 109.5)			53 (43.5, 63.5)			34 (29, 37)		
Better	128 (42.5)	103.5 (86, 116)			56 (44, 64.75)			35 (32, 41)		
Good	43 (14.3)	103 (73, 119)			59 (44, 68)			37 (31, 41)		

Z, Mann–Whitney U Test; H, Kruskal-Wallis H Test; *P* < 0.05, indicates statistically significant differences between groups; The scores of the three scales represent, M (P_25_, P_75_).

### 3.2 Correlation analysis of active aging, coping strategies, and social support among older adults patients with chronic diseases

Social support among older adults with chronic diseases was significantly positively correlated with medical coping strategies (*r* = 0.408, *p* < 0.01) and with active aging (*r* = 0.422, *p* < 0.01). Furthermore, active aging was also significantly positively correlated with medical coping strategies (*r* = 0.485, *p* < 0.01).

### 3.3 Common method bias

Harman’s single-factor test was applied to assess common method bias. This approach has inherent limitations, as it provides only a preliminary evaluation and cannot fully exclude potential common method bias. Anonymous questionnaires were used to reduce response bias. The results showed that 13 factors had eigenvalues greater than 1, and the first factor accounted for 28.771% of the total variance, which is below the critical threshold of 40%. Therefore, no serious common method bias was detected, and the data reliability was considered acceptable.

### 3.4 Mediating effect test

**Figure 1:**
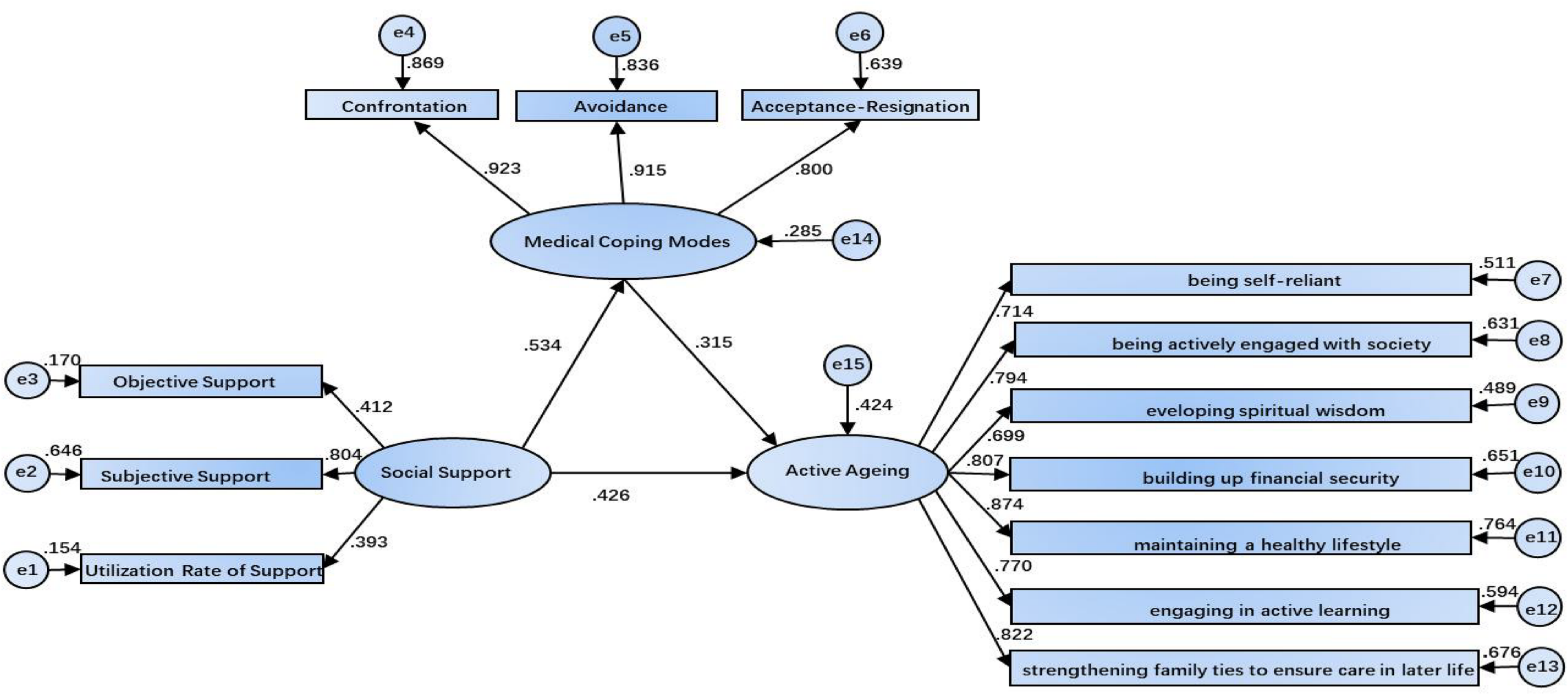
Mediating lmodel of medical coping strategies in the relationship between social support and active aging in older adults patients with chronic diseases. 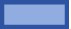: Observed variable; 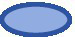: Latent variable; 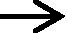: Direct effect path between variables; The arrow points in the direction of influence; The value on the arrow is the standardized path coefficient (*β*), which reflects the strength and direction of the influence: 1. Social support → medical coping strategies (β = 0.534), 2. Medical coping strategies → active aging (*β* = 0.315), 3. Social support → active aging (*β* = 0.426). e1–e15: Residual term (error term), whose side values are standardized estimates of residual variance, representing the degree of variation of the corresponding variable that has not been explained by the model. This model does not estimate the correlation between variables and error terms, only analyzes the one-way direct and mediating effects between variables.

To assess the mediating effect, a structural equation model was constructed in AMOS 24.0, with social support as the independent variable, medical coping as the mediator, and active aging as the dependent variable. The model was initially estimated using maximum likelihood estimation and subsequently refined. After modification, the model demonstrated acceptable fit indices: the χ^2^/df was 2.75, below the recommended threshold of 3; the GFI = 0.912, NFI = 0.928, TLI = 0.940, IFI = 0.953, and CFI = 0.953, all of which fell within the acceptable range (>0.9); and the RMSEA = 0.076, below the critical value of 0.08. These results indicate a well-fitting mediation model, illustrating the role of medical coping strategies in the relationship between social support and active aging among older adults with chronic diseases, as depicted in Figure 1.

All path coefficients in the structural equation model were statistically significant (*p* < 0.05). Specifically, social support had a significant positive effect on medical coping (*β* = 0.534, *p* < 0.001), and medical coping in turn exerted a significant positive influence on active aging (β = 0.315, *p* < 0.001). Simultaneously, social support had a significant direct positive effect on active aging (*β* = 0.426, *p* < 0.001). Mediation effects were further examined using the Bootstrap method with 5,000 resamples and a 95% confidence interval (CI) (Tables 2, 3). The results indicated that the direct effect of social support on active aging was 0.426 (95% CI: 0.248–0.634), accounting for 71.72% of the total effect. The indirect effect of medical coping as a mediator was 0.168 (0.534 × 0.315, 95% CI: 0.076–0.295), representing 28.28% of the total effect, with the 95% CI not including zero. These findings suggest that medical coping, particularly the confrontation coping style, partially mediates the relationship between social support and active aging. Detailed results are presented in Tables 2, 3.

**Table 2:** Bootstrap analysis of the mediating role of medical coping on social support and active aging among older adults chronic disease patients.

Structural paths	Items	Effect	SE	95%CI		Effect value%
				Lower	Upper	
Social support→Active aging	Total effect	0.594	0.072	0.439	0.73	/
Social support→Active aging	Direct effect	0.426	0.102	0.248	0.634	71.72
Social support→Coping style→Active aging	Indirection effect	0.168	0.055	0.076	0.295	28.28

SE, standard error; CI, confidence interval.

**Table 3:** Multivariate linear regression analysis of coping strategies’ impact on active aging levels.

Variability	***β***	Sig
Coping style (Confrontational)	0.474	0.000
Coping style (Submissive)	0.147	0.143
Coping style (Avoidant)	−0.116	0.142

*β*, standardized regression coefficient; Sig, significance level.

## 4 Discussion

This study systematically examined the current status of active aging among older adults with chronic diseases, explored the relationships between social support and coping styles, and verified the mediating role of medical coping strategies using structural equation modeling. The findings carry important theoretical and practical implications. Age-related declines in physiological function contribute to a marked increase in the prevalence of multimorbidity among the older adults (1). Analysis of demographic variables further indicated that participants covered by employee medical insurance exhibited higher scores in both active aging and social support. This may be attributed to the more comprehensive medical coverage and greater accessibility to healthcare resources enjoyed by this group, which likely enhances their perception of social support and helps maintain relatively better health status (18). Consequently, when confronted with health challenges, these individuals may adopt coping strategies more effectively, increasing their likelihood of engaging in activities associated with active aging. Active aging scores were significantly higher in the high-income group than in the low-income group (*p* = 0.006). This difference was not primarily driven by the direct effect of economic conditions. Adequate financial resources may provide essential support for older adults with chronic diseases to adopt confrontational coping strategies, thereby indirectly enhancing active aging. Conversely, older adults with multimorbidity and substantial economic burdens may avoid hospitalization due to the inability to afford medical expenses (19). This behavior reflects passive coping strategies, such as acquiescence or avoidance, which can undermine effective chronic disease management, accelerate disease progression, and ultimately reduce levels of active aging (18, 20). Moreover, widowed individuals, those living alone, those lacking regular exercise habits, or those without children exhibited lower scores in both social support and active aging. According to the Social Convoy Model, spouses and children constitute the innermost and most stable core of an individual’s social support network (21). Widowhood disrupts this core, depriving the older adults of emotional attachment and daily life support, and may lead to contraction of the broader social network previously centered on the marital relationship (22, 23). The absence of children further diminishes available care and support, leaving older adults without direct, reliable resources to cope with health crises and long-term care needs. This pattern aligns with the traditional Chinese filial piety culture, which emphasizes that children should provide both material and emotional support to their parents (19, 24). Living alone exacerbates social isolation, reducing opportunities to access external support from friends or neighbors. Social support serves as a psychological foundation that enables older adults to adopt effective coping strategies by improving mental health and alleviating loneliness, thereby facilitating active aging (25–27). Additionally, a lack of regular exercise limits key avenues for building and reinforcing extended social support, such as engagement with community activities or interest groups. Physical activity, including square dancing and jogging, has been shown to promote active aging among middle-aged and older adults by maintaining social interactions and creating opportunities to form new friendships (6, 28).

From the perspective of variable associations, social support was significantly positively correlated with active aging ( *r* = 0.422, *p* < 0.01), consistent with previous findings (29). As an essential external resource for coping with stress, social support encompasses emotional, material, and informational dimensions (26). Specifically, emotional care from family members is associated with reduced feelings of loneliness and anxiety related to patients’ illnesses, thereby helping maintain patients’ psychological well-being (30). Health guidance from community and medical institutions is related to disease management and physiological function maintenance, while interactions with friends and social groups are associated with social participation (31). These associations indicate a relationship between social support and active aging. Additionally, social support was positively correlated with coping strategies ( *r* = 0.408, *p* < 0.01), suggesting that sufficient social support may encourage patients to adopt active medical coping strategies (32, 33). According to coping theory, patients who perceive adequate social support may have stronger confidence in managing their diseases and tend to choose positive coping methods (e.g., confronting issues or seeking help), rather than negative strategies (e.g., avoidance or surrender). This aligns with the current findings. Family encouragement is associated with active treatment cooperation and regular medical reviews. Disease management information from community healthcare centers helps patients develop scientific understanding and optimize coping behaviors (34). In contrast, coping methods in Western countries may rely more on problem-oriented individual interventions (e.g., seeking psychological counseling or joining patient support groups), rather than family-driven confrontational approaches (35). Therefore, the indirect effect of social support on active aging via coping strategies might be weaker, and other mediators (e.g., health literacy or self-efficacy) may exist.

Second, Western countries may rely more on professional support and prefer emotion-focused coping. Due to differences in medical welfare, East Asian countries with similar cultures may differ in the strength of this association. Community-based patients often have milder conditions and require support focused on long-term rehabilitation guidance. Their coping strategies emphasize self-management, and the influence of social support on active coping may be weaker than for hospitalized patients (36, 37). Additionally, active aging was significantly positively correlated with medical coping strategies (*r* = 0.485, *p* < 0.01), indicating that positive medical coping is a key intrinsic factor in promoting active aging among older adults with chronic diseases. When these patients adopt a proactive approach to their illness, such as actively acquiring knowledge about their condition and adhering to rehabilitation exercises, they can effectively slow disease progression, mitigate the impact of disease on daily functioning, and maintain a high quality of life and social participation (29). In contrast, negative coping strategies often result in poor disease management, exacerbate psychological distress, and ultimately reduce levels of active aging. Further analysis revealed a significant positive correlation between active aging scores and the confrontation coping style (*r* = 0.513, *p* < 0.01), suggesting that higher active aging is associated with a greater tendency to employ confronting coping strategies.

The mediation analysis demonstrated that medical coping strategies partially mediated the relationship between social support and active aging. The direct effect accounted for 71.72% of the total effect, while the indirect effect accounted for 28.28%, and the model exhibited good fit (χ^2^/df = 2.75, RMSEA < 0.08, with fit indices such as GFI and CFI meeting recommended standards). The relatively large proportion of the direct effect suggests that social support can directly provide emotional, material, and service-related resources to older adults with chronic diseases, creating favorable conditions for adopting appropriate coping strategies. By enhancing patients’ medical coping behaviors, social support also indirectly promotes active aging, further supporting the validity of the proposed “social support → coping strategy → active aging” pathway.

This study has several limitations. First, a cross-sectional survey design was used, which cannot establish causal relationships among variables. Second, the sample source may have regional limitations, and the generalizability of results requires further validation. Third, potential confounding factors, such as disease severity and educational level, were not considered. In the future, cross-cultural studies involving diverse healthcare systems and populations could be conducted to enhance the universality of the findings.

## 5 Conclusion

Medical coping strategies were found to partially mediate the relationship between social support and active aging in older adults with chronic diseases. Specifically, 28.28% of the effect of social support on active aging was transmitted indirectly through coping strategies. Among these, the confrontation-oriented coping style emerged as the central component of the mediating pathway and demonstrated a significant positive predictive effect on active aging. To promote higher levels of active aging in this population, it is crucial to both strengthen social support systems, through measures such as enhancing medical security and reinforcing family and community support, and to guide patients toward adopting confrontation-based positive coping strategies. The combined implementation of these approaches can more effectively facilitate the attainment of active aging among older adults individuals with chronic conditions.

## Data Availability

The raw data supporting the conclusions of this article will be made available by the authors, without undue reservation.
